# Transmission Dynamics of Resistant Bacteria in a Predator-Prey System

**DOI:** 10.1155/2015/638074

**Published:** 2015-03-04

**Authors:** Xubin Gao, Qiuhui Pan, Mingfeng He

**Affiliations:** ^1^School of Mathematical Sciences, Dalian University of Technology, Dalian 116024, China; ^2^City institute, Dalian University of Technology, Dalian 116600, China; ^3^School of Innovation Experiment, Dalian University of Technology, Dalian 116024, China

## Abstract

This paper discusses the impact on human health caused by the addition of antibiotics in the feed of food animals. We use the established transmission rule of resistant bacteria and combine it with a predator-prey system to determine a differential equations model. The equations have three steady equilibrium points corresponding to three population dynamics states under the influence of resistant bacteria. In order to quantitatively analyze the stability of the equilibrium points, we focused on the basic reproduction numbers. Then, both the local and global stability of the equilibrium points were quantitatively analyzed by using essential mathematical methods. Numerical results are provided to relate our model properties to some interesting biological cases. Finally, we discuss the effect of the two main parameters of the model, the proportion of antibiotics added to feed and the predation rate, and estimate the human health impacts related to the amount of feed antibiotics used. We further propose an approach for the prevention of the large-scale spread of resistant bacteria and illustrate the necessity of controlling the amount of in-feed antibiotics used.

## 1. Introduction

As the world's population continues to increase, methods for improving the production of livestock have become a great challenge. The growth-promoting effects of antibiotics were first discovered in the 1940s when chickens fed by-products of tetracycline fermentation were found to grow faster than those not fed with these by-products [1]. Since then, many antimicrobials have been found to improve average daily weight gain and feed efficiency in livestock in a variety of applications [2–4], and this process has come to be known as “growth promotion.” Antibiotics used in feeding not only increase production but can also prevent disease. Infected animals will not only affect the production capacity, but as the stocking density increases, infection is more likely to be transmitted at a faster rate. Therefore, the most effective strategy to prevent epidemics in livestock is to add antibiotics to the animal feed, which has become common practice [5].

Although the use of antibiotics as feed additives can promote animal growth, there are also various side effects that affect the human consumer. When antibiotics are used in animal feed, resistance is likely to be selected for in the normal and infected bodies, and therefore increase in prevalence in the population [6–10]. Furthermore, Perreten et al. [11] demonstrated that resistant bacteria are highly contagious, and resistance can transfer to other strains through plasmids or by direct contact. Therefore, other congeneric animals can readily and rapidly become infected with antibiotic-resistant bacteria.

Resistant bacteria not only transmit horizontally but can also transmit across species. When animals harboring antibiotic-resistant bacteria come in close contact with humans or are consumed by humans, the resistant bacteria may transfer to the humans [12].

Harwood et al. [13] confirmed that resistant bacteria could spread through sewage to reach the general environment. Consequently, if birds or other types of urban wildlife were to drink the sewage, the resistant bacteria would spread widely either through their feces or if directly consumed by other animals. Resistant bacteria have been found in wild rodents and in some domestic animals [14, 15]. Direct contact is another important way that resistant bacteria can transfer from animals to humans. Van den Bogaard et al. [16] and Donabedian et al. [17] conducted experiments that demonstrated that resistant bacteria do indeed have the ability of being transmitted from animals to humans. Anderson et al. [18] indicated that different types of resistant bacteria present in the food chain have different effects on humans. In a review, Phillips et al. [19] explained that the use of antibiotics in food animals poses a risk to human health and listed the various ways that resistant bacteria can transfer from animals to humans through the food chain and by other means. Tollefson and Miller [20] described the history of antimicrobial use and regulation in animals, the related public health concern, the current animal drug approval process in the United States, the international perspective, and the Food and Drug Administration's (FDA's) proposed procedures for evaluating the human health impact of the antimicrobial effects associated with animal drugs intended for use in food-producing animals. All of the articles cited above clearly show that resistant bacteria can indeed transfer from food animals to humans through direct contact or consumption.

Once humans are infected with resistant bacteria, the bacteria will readily transfer among the human population. In order to prevent the wide spread of resistant bacteria among humans, many researchers have studied the spread process of resistant bacteria between humans in the hospital and have proposed prevention measures [21–29]. However, almost no quantitative analysis of the spread of resistant bacteria from animals to humans has been performed to date. In particular, no study has yet focused on the process of transmission through the food chain, in which food animals consume large amounts of antibiotic resistant bacteria and then transmit the resistant bacteria to humans through direct contact or predation, and the bacteria continue to spread among humans. In this paper, we discuss this process and report the results of a quantitative analysis of the effects that the amount of antibiotics used as feed additive have for human health.

Based on the resistant bacteria transmission rule, we use Lotka–Volterra equations [30] to derive a model of the spread of resistant bacteria in a predator-prey system. Resistant bacteria can be viewed as an infectious disease in a predator-prey system [31–36]. The predator-prey systems are key factors in ecological systems. So many articles have focused on the spread of infectious diseases in a predator-prey system [37–43]. Some researchers have adopted a predator-prey model with only the prey affected by disease and some of them are representative: Anderson and May [44], Chattopadhyay et al. [35, 43], Xiao and Chen [45], Greenhalgh and Haque [46], and Sinha et al. [47–52], whereas others have modeled disease in only the predator, for instants, Haque and Venturino [53, 54], Hilker and Schmitz [55], and Auger et al. [56]. Few studies consider the predator-prey models with disease in both predator and prey. Hsieh and Hsiao [57], Pada Das et al. [58], and Gao et al. [59] modeled the situation in which both the predator and the prey have the disease, but in the first article, the disease was not contagious among the predators, and in the second article, there was no cross-infection, and in the third article, the diseases were transmitted vertically in both populations. Briggs and Hoopes [60], Chaudhuri et al. [61, 62] discussed cross-infection model. Furthermore, two other models [63, 64] were presented in which the disease could cross the species barrier in an interacting population. We here present a model in which the infected disease is represented by resistant bacteria that can be transmitted between a prey and a predator in both directions through contact or consumption.

This paper is organized in the following manner. Based on the available evidence demonstrating that resistant bacteria can spread between different populations, we establish differential equations model in Section 2. In Section 3, we derive the equilibrium points, and discuss the nonnegative points' existence conditions. In Section 4, we analyze the basic reproduction numbers. In this section, the threshold parameters are related to meaningful biological conditions. We then determine the stability conditions of the equilibrium points and prove them. In Section 5, we provide numerical results to discuss some interesting biological cases that our model is able to exhibit and the role of some key parameters in the system. We conclude by discussing the practical significance and application of the model.

## 2. Model

In this model, food animals consume antibiotic-containing feed over an extended period of time, and the bacteria evolve resistance to the antibiotics. This resistance can spread to humans via consumption or direct contact and then spread among the human population. Thus, both food animals and humans can be infected by the resistant bacteria. There are four populations in our model: *x*_1_(*t*) is the population size of the susceptible food animals at time *t*. *x*_2_(*t*) is the population size of the infected food animals at time *t*. *y*_1_(*t*) represents the amount of susceptible humans at time *t*, and *y*_2_(*t*) represents the amount of infected humans at time *t*.

To construct the model, we made the following assumptions.

1. There is no recovery or immunity from resistant bacteria.
2. The resistant bacteria can be transmitted vertically.
3. *b* is the growth rate of the food animals; *K* is the carrying capacity of the food animals.
4. *β*_1_ (0 < *β*_1_ < 1) represents the conversion rate from susceptible food animals to infected animals. Since the quantities of the antibiotics added in feed are usually stationary, the conversion rate from susceptible food animals to infected animals is constant. The value of *β*_1_ is only related to antibiotics in feed additives and is not related to *x*_2_. To some extent, *β*_1_ also represents the proportion of antibiotics in the feed. In this model, we do not consider the resistant bacteria's spread among food animals.
5. To determine whether or not the food animals are infected is nearly impossible; however, resistance in the food animals does not affect the predation rate *p*. Humans reproduce at a certain rate after eating: *ep* is the predator's reproduction rate, where 0 < *e* < 1.
6. *d* is the death rate of infected animals; *d*_1_ is the natural death rate of humans; *d*_2_ is the human death rate due to infection by resistant bacteria, *d*_2_ > *d*_1_.
7. *β*_2_ is the infection rate among humans and *β*_3_ (0 < *β*_3_ < 1) is the infection rate from food animals to humans. Due to the cross-infection mechanism of resistant bacteria, the term *β*_3_ *x*_2_ *y*_1_ characterizes the transmission dynamics [63, 64]. The resistant bacteria's transmission in food animals is not considered.

Given the assumptions listed above, we obtain the following autonomous nonlinear differential equations:

(1) x˙1=bx1(1−x1+x2K)−bβ1x1−px1y1−px1y2,x˙2=bβ1x1−px2y1−px2y2−dx2,y˙1=−d1y1−β2y1y2+epx1y1+epx2y1−β3x2y1,y˙2=−d2y2+β2y1y2+epx1y2+epx2y2+β3x2y1.

To analyze the stability of the system, we first determine the equilibrium points.

## 3. Equilibrium Points

### 3.1. The Existence of Nonnegative Equilibrium Points

It is easy to obtain the four nonnegative equilibrium points of model (1). They are

(2) E0(0,0,0,0),E1(Kd1−β1d+bβ1,Kβ1b1−β1d+bβ1,0,0),E2x~1,x~2,0,y~2,E3(x1∗,x2∗,y1∗,y2∗),

where

(3) x~1=d2(Kepd+Kepb−d2b−Kepbβ1)(ep(Kepd+Kepb−d2b)),x~2=Kβ1d2b(Kepd+Kepb−d2b),y~2=b(Kep−Kepβ1−d2)(ep2K),x1∗=XKd+Kb−bX−bβ1KbX+Kβ1,x2∗=X,y1∗=Tpβ2bX+Kβ1,y2∗=−Xd1b−β3X2b−bd1β1K+KepXd   −Kβ1β3Xb+bKepXx2  ·β2bX+Kβ1−1,T=β2b2β1K+pXd1b+pX2β3b+pbβ1d1K  −ep2XKd+pXKβ1β3b−ep2XKb  −Xdβ2b−Xb2β1β2−b2β12β2K,

and *X* is the real positive root of the equation

(4) $$\begin{array}{c} H_{1}x^{3}+H_{2}x^{2}+H_{3}x+H_{4}=0, \end{array}$$

where

(5) H1=pβ3bd1−d2,H2=ep2d2bK+β2epdbK−ep2d1Kd−β2dd1bhhhh+β2epd2K−pd2bd1−d1b2β1β2hhhh+ep2d2Kd−2pd2Kbβ1β3+2pd1Kbβ1β3hhhh−ep2d1bK+pd12b+β2b2β1epK+β2bβ1epKd,H3=−2d1b2β12β2K+ep2d2K2β1b−ep2d1K2β1dhhhh−ep2d1K2β1b+ep2d2K2β1d−2pd2bβ1d1Khhhh−β2dd1bβ1K+2pd12bβ1K+pd1K2β12β3bhhhh−β2epb2K2β1−epK2dbβ1β2+β2b2β1Kd1hhhh+β2β12epK2bd−pd2K2bβ12β3+β2b2β12epK2,H4=pd12K2β12b+β2b2β12K2d1hhhh−β2b2β13K2d1−pd2K2β12bd1.

In the next section, we will discuss the stability conditions of the different situations.

## 4. Qualitative and Quantitative Analysis of the Model

For each equilibrium point, we focus on whether the susceptible human can survive.

### 4.1. Qualitative Analysis of Model (1)

First, we discuss the biological significance of the three threshold parameters that could be obtained from the stability analysis of the boundary equilibrium points. We will discuss the basic reproduction numbers in the following.

We define

(6) R01=epK1−β1d+bβ1d1d+bd1β1+bβ1Kβ3−bKβ3β12,R02=epK1−β1d2,R03=epx~1+x~2d1+β2y~2+β3x~2,

where ${\tilde{x}}_{1}$, ${\tilde{x}}_{2}$, and ${\tilde{y}}_{2}$ are as defined in Section 3.

Each of the threshold parameters has a clear and distinct biological meaning.

For *E*_1_, the susceptible human growth rate is *epK*(1 − *β*_1_) and the mortality rate is (*d*_1_ *d* + *bd*_1_ *β*_1_ + *bβ*_1_ *Kβ*_3_ − *bKβ*_3_ *β*_1_^2^)/(*d* + *bβ*_1_), whereas the infected human growth rate is *epK*(1 − *β*_1_) and the mortality rate is *d*_2_.

*R*_0_^1^ < 1 implies that the susceptible predators will become extinct, while *R*_0_^2^ < 1 implies that the infected predators will become extinct. Hence, the combination of these two conditions results in *E*_1_ being locally asymptotically stable.

For *E*_2_, the susceptible human growth rate is $ep({\tilde{x}}_{1}+{\tilde{x}}_{2})$ and the mortality rate is $\left(d_{1}+\beta_{2}{\tilde{y}}_{2}+\beta_{3}{\tilde{x}}_{2}\right)$.

*R*_0_^2^ > 1 means that the infected predator population will exist.

*R*_0_^3^ < 1 implies that the susceptible predator population will become extinct. Hence, the combination of these two conditions results in *E*_2_ being locally asymptotically stable. Similarly, *R*_0_^2^ > 1 and *R*_0_^3^ > 1 are the locally asymptotically stable conditions for *E*_3_.

These conclusions are summarized in Table 1.

### 4.2. Quantitative Analysis of Model (1)

In order to determine the stability conditions of the equilibrium points, we calculated the Jacobi matrix *J* = (*J*_*ij*_)_4×4_ for the equations, where

(7) J11=b(1−x1+x2K)−bx1K−bβ1−py1−py2,J12=−bx1K,  J13=−px1,  J14=−px1,J21=bβ1,  J22=−py1−py2−d,J23=−px2,  J24=−px2,J31=epy1,  J32=epy1−β3y1,J33=−d1−β2y2+epx1+epx2−β3x2,J34=−β2y1,  J41=epy2,  J42=epy2+β3y1,J43=β2y2+β3x2,  J44=−d2+β2y1+epx1+epx2.

### 4.3. Stability of the Boundary Equilibrium Point, *E*_0_

**Theorem 1 .**

The boundary equilibrium point *E*_0_(0,0, 0,0) is always unstable.

**Proof of Theorem 1.**

We obtain the Jacobi matrix for *E*_0_(0,0, 0,0) as

(8) $$\begin{array}{c} J_{0}=\left[\begin{array}{cccc} b-b\beta_{1} & 0 & 0 & 0\\ b\beta_{1} & 0 & 0 & 0\\ 0 & 0 & -d & 0\\ 0 & 0 & 0 & -d_{2} \end{array}\right]. \end{array}$$

The eigenvalues of *J*_0_ are

(9) λ1=0,  λ2=b−bβ1,λ3=−d,  λ4=−d2.

As a result of *λ*_2_ = *b* − *bβ*_1_ > 0, the boundary equilibrium point *E*_0_(0,0, 0,0) is always unstable.

### 4.4. Stability of the Boundary Equilibrium Point, *E*_1_

**Theorem 2 .**

If *R*_0_^1^ < 1; *R*_0_^2^ < 1, the boundary equilibrium point *E*_1_(*Kd*(1 − *β*_1_)/(*d* + *bβ*_1_), *Kβ*_1_ *b*(1 − *β*_1_)/(*d* + *bβ*_1_), 0,0) is locally asymptotically stable.

The proof of Theorem 2 is provided in the Appendix A.

### 4.5. Stability of the Boundary Equilibrium Point, *E*_2_

**Theorem 3 .**

If *R*_0_^2^ > 1 and *R*_0_^3^ < 1, the boundary equilibrium point $E_{2}({\tilde{x}}_{1},{\tilde{x}}_{2},0,{\tilde{y}}_{2})$ is locally asymptotically stable, where ${\tilde{x}}_{1}$, ${\tilde{x}}_{2}$, and ${\tilde{y}}_{2}$ are as defined in Section 3.

The proof of Theorem 3 is provided in the Appendix B.

### 4.6. Stability of the Interior Equilibrium Point, *E*_3_

**Propose 1 .**

If *R*_0_^2^ > 1 and *R*_0_^3^ > 1, the interior equilibrium point *E*_3_(*x*_1_^*^, *x*_2_^*^, *y*_1_^*^, *y*_2_^*^) is locally asymptotically stable.

We only numerically investigated the system's behavior around the interior feasible equilibrium point *E*_3_ and provide the necessary numerical proof in the next section.

### 4.7. Periodic Orbit of Model (1)

**Theorem 4 .**

No periodic orbit of system (1) exists in

(10) $$\begin{array}{c} \Omega =\left\{\left(x_{1},x_{2},y_{1},y_{2}\right)\;|\;x_{1}>0,x_{2}>0,y_{1}>0,y_{2}>0\right\}\subset R^{4}. \end{array}$$

The proof of Theorem 4 is provided in the Appendix C.

## 5. Numerical Results and Parameter Analysis

### 5.1. Numerical Results

Based on the analysis in the section above, conversion rate *β*_1_ and predation rate *p* that are the key parameters not only can affect all the basic reproduction numbers but also can be controlled by human. *β*_1_  (0 < *β*_1_ < 1) represents the conversion rate from susceptible food animals to infected animals. In this paper, the conversion from susceptible food animals to infected animals is only due to the addition of antibiotics in the feed of food animals. In another word, even though without contacting, susceptible food animals can also convert to infected animals by eating feed with antibiotics. Therefore, the conversion rate *β*_1_ depends on the amount of antibiotics used in feed. For the same feed, the amount of antibiotics added is larger, there will be more susceptible animals that convert to infected animals, and the conversion rate is bigger.

For further analysis of the steady-state of the equilibrium points and the parameter effects of *β*_1_ and *p*, we illustrate some key numerical solutions.

When the parameter values are fixed at *b* = 0.8, *K* = 15, *p* = 0.45, *d*_1_ = 0.02, *β*_2_ = 0.005, *e* = 0.05, *d*_2_ = 0.07, *d* = 0.01, *β*_1_ = 0.1, and *β*_3_ = 0.08, we can calculate all of the equilibrium points as

(11) E11.5,12,0,0,E22.72,0.39,0,1.23,E31.46,0.18,0.93,0.48.

The basic reproduction numbers are *R*_0_^2^ = 4.34 > 1, *R*_0_^3^ = 1.23 > 1, and as seen in Propose 1, the equilibrium point *E*_3_(1.46,0.18,0.93,0.48) is locally asymptotically stable.

The numerical proof of Propose 1 is as follows.

For the interior equilibrium point *E*_3_(1.46,0.18,0.93,0.48), the Jacobi matrix is

(12) $$\begin{array}{c} J_{3}^{*}=\left[\begin{array}{cccc} -0.08 & -0.08 & -0.66 & -0.66\\ 0.02 & -0.05 & 0 & -0.005\\ 0.01 & 0.09 & 0.02 & -0.03\\ 0.31 & 0.36 & 0.46 & -0.01 \end{array}\right]. \end{array}$$

The eigenvalues of *J*_3_^*^ are *λ*_1,2_ = −0.05 ± 0.15*i*, *λ*_3_ = −0.63, and *λ*_4_ = −0.05, and all the real parts of the eigenvalues are negative. Hence, the Routh-Hurwitz criteria are satisfied. Therefore, *E*_3_(1.46,0.18,0.93,0.48) is locally asymptotically stable.

### 5.2. Role of the Parameter *β*_1_ in Model (1)

*β*_1_   (0 < *β*_1_ < 1) represents the conversion rate from susceptible food animals to infected animals and also represents the proportion of antibiotics in the feed. Therefore, as *β*_1_ plays a major role in the outcome of the model, we will discuss its effect on the system. For convenience, our numerical results are summarized in Table 2. The following initial values are used: *x*_1_(0) = 0.5, *x*_2_(0) = 0.4, and *y*_1_(0) = 1, *y*_2_(0) = 2.

In order to clearly show population dynamics for each case, three numerical results are given in the following for different values of *β*_1_.

**Figure 1:**
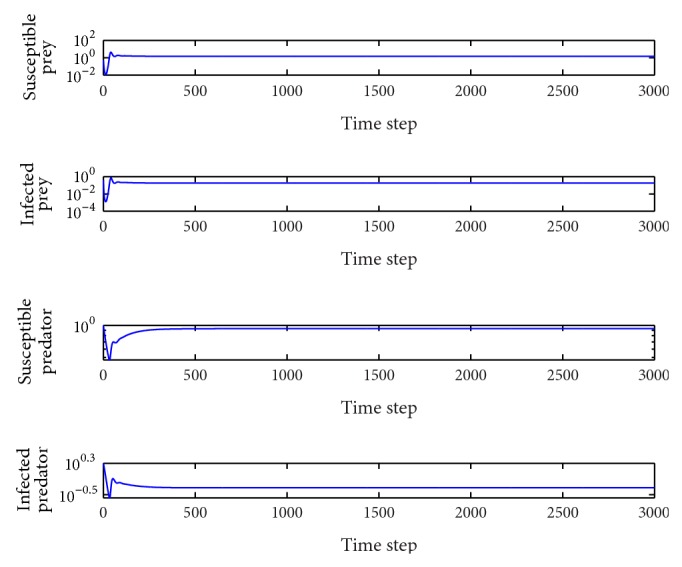
Evolution of the system populations for *β*_1_ = 0.1; other parameters are as defined in Table 2.

**Figure 2:**
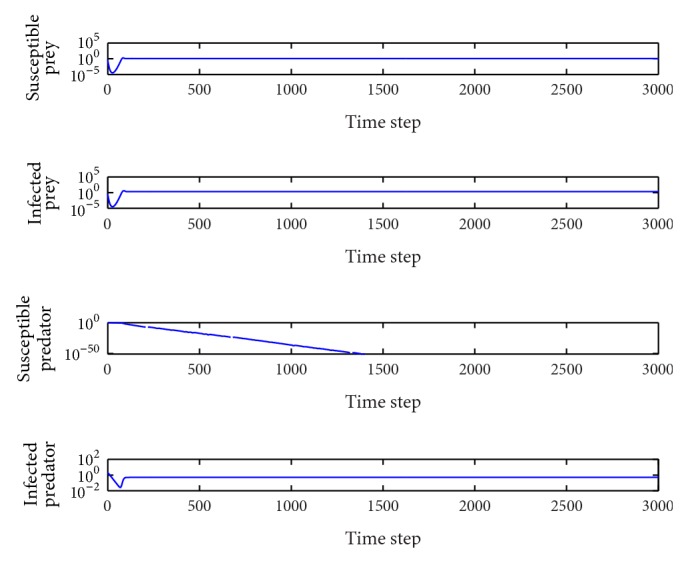
Evolution of the system populations for *β*_1_ = 0.5; other parameters are as defined in Table 2.

**Figure 3:**
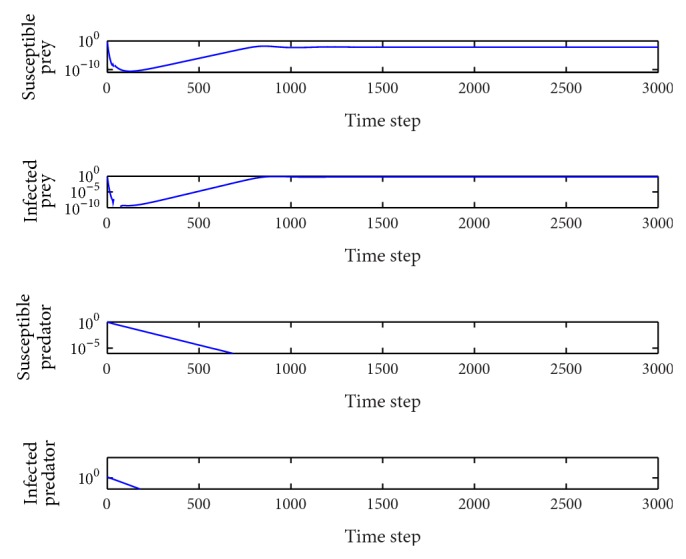
Evolution of the system populations for *β*_1_ = 0.96; other parameters are as defined in Table 2.

- *β*_1_ = 0.1.
- In this case, the basic reproduction number is *R*_0_^2^ = 4.34 > 1, *R*_0_^3^ = 1.23 > 1 and so the interior equilibrium point *E*_3_(1.46,0.18,0.93,0.48) is locally asymptotically stable (the proof is provided above). The system tends toward the coexistence equilibrium (Figure 1). When increasing *β*_1_ from zero to 0.14, there is only a quantitative change, and the system's qualitative behavior remains the same. At this time, all the species coexist.
- *β*_1_ = 0.5.
- In this case, the basic reproduction number is *R*_0_^2^ = 2.41 > 1, *R*_0_^3^ = 0.40 < 1 and so the equilibrium point *E*_2_(1.18,1.93,0, 0.52) is locally asymptotically stable (Figure 2). The susceptible humans die out and the other three population types coexist in the system. This behavior remains for 0.14 < *β*_1_ < 0.79.
- *β*_1_ = 0.96.
- In this case, we can calculate the basic reproduction number as *R*_0_^1^ = 0.20 < 1, *R*_0_^2^ = 0.19 < 1 and so the equilibrium *E*_1_(0.008,0.59,0, 0) is locally asymptotically stable. Both of the human populations are extinct, and there are only food animals remaining in the system (Figure 3). In the wide range 0.79 < *β*_1_ < 1, the behavior of the system is qualitatively the same.

### 5.3. Role of the Parameter *p* in Model (1)

Besides the parameter *β*_1_, we are also concerned about the role of the parameter *p* in the system. *p* is the predation rate in the model (1). As a result of the predator reproduction is dependent on its predation rate, so the change of predation rate affected both prey and predator. Owing to the predation rate can be controlled artificially, so predation rate is also a key parameter we care about. In this section, we will discuss the role of parameter *p* in model (1). The conclusions are summarized in Table 3. The initial value are *x*_1_(0) = 0.5, *x*_2_(0) = 0.4, *y*_1_(0) = 1, and *y*_2_(0) = 2, and the parameter values except *p* are *b* = 0.8, *K* = 15, *β*_1_ = 0.1, *d*_1_ = 0.02, *β*_2_ = 0.005, *e* = 0.05, *d*_2_ = 0.07, *d* = 0.01, and *β*_3_ = 0.08.

We describe the influence of predation rate on the system under three different situations.

**Figure 4:**
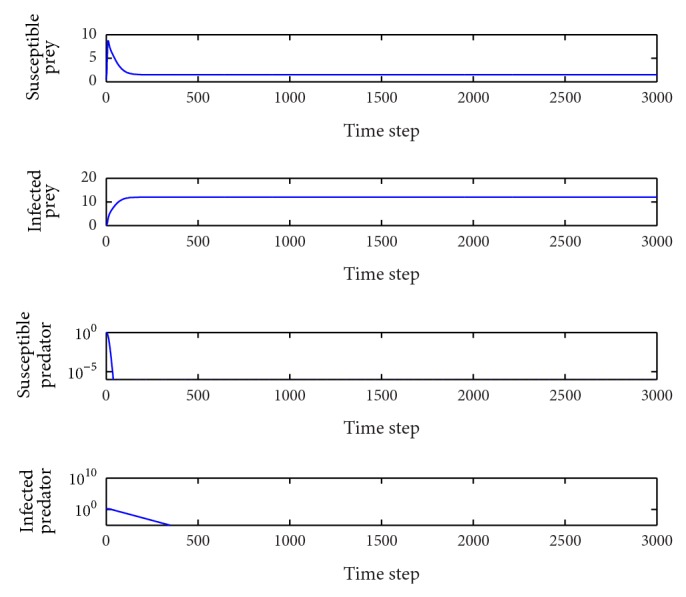
Evolution of the system populations for *p* = 0.05; other parameters are as defined in Table 3.

**Figure 5:**
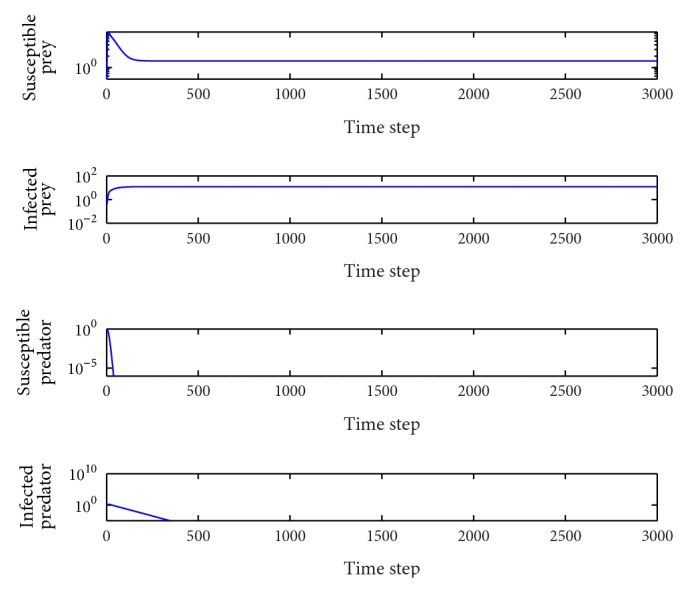
Evolution of the system populations for *p* = 0.25; other parameters are as defined in Table 3.

**Figure 6:**
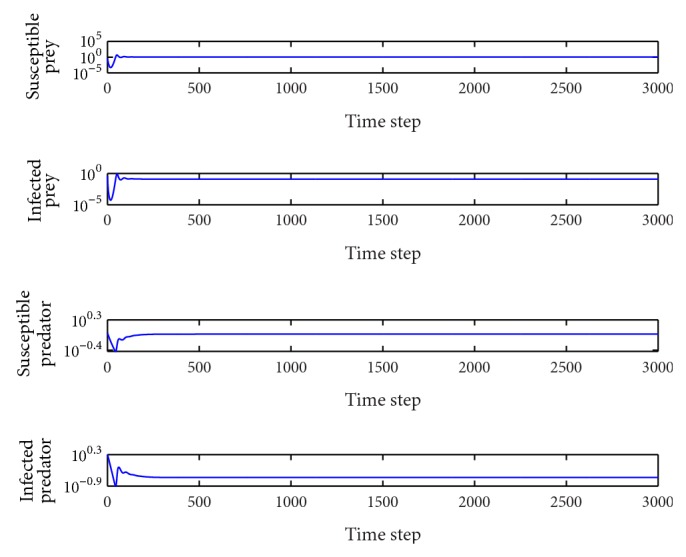
Evolution of the system populations for *p* = 0.55; other parameters are as defined in Table 3.

- *p* = 0.05.
- In this case, the basic reproduction number are *R*_0_^1^ = 0.03 < 1, *R*_0_^2^ = 0.48 < 1, and the equilibrium *E*_1_(1.5,12,0, 0) is locally asymptotically stable. Both of the human populations are extinct, and there are only food animals remaining in the system (Figure 4). It is similar to the situation in Figure 3. In the wide range 0 < *p* < 0.104, the behavior of the system is qualitatively the same.
- *p* = 0.25.
- In this case, the basic reproduction numbers are *R*_0_^2^ = 2.41 > 1, *R*_0_^3^ = 0.71 < 1 and so the equilibrium point *E*_2_(1.18,1.93,0, 0.52) is locally asymptotically stable (Figure 5). It is similar to the situation in Figure 2. The susceptible humans die out and the other three population types coexist in the system. This behavior remains for 0.104 < *p* < 0.351.
- *p* = 0.55.
- In this case, the basic reproduction numbers are *R*_0_^2^ = 5.30 > 1, *R*_0_^3^ = 1.41 > 1 and so the interior equilibrium point *E*_3_(1.00,0.12,0.94,0.26) is locally asymptotically stable. The system tends toward the coexistence equilibrium (Figure 6). It is similar to the situation in Figure 1. When increasing *p* from 0.351 to 1, there is only a quantitative change, and the system's qualitative behavior remains the same. At this time, all the species coexist.

### 5.4. Role of the Parameters *β*_1_ and *p* in the System

**Figure 7:**
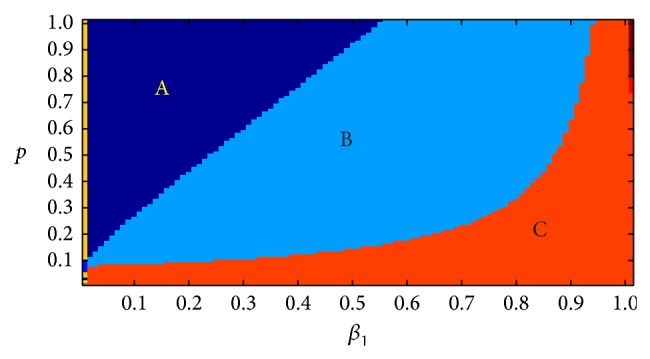
Changing *β*_1_ and *p* while maintaining the other parameters and the initial values fixed as defined in Table 3, three regions were obtained after 3000 time steps.

In the following section, we discuss the effect of *β*_1_ and *p* together, while maintaining the other parameters fixed as defined in Table 3. We observed the dynamics when changing the parameters *β*_1_ and *p* between 0 and 1. The resulting image is shown in Figure 7, in which three different regions were obtained after 3000 time steps, which are indicated by different colors.

In region A, four populations can coexist. In region B, the susceptible humans die out, and the other populations coexist. In region C, there are only food animals in the system.

Based on the conclusion shown in Table 1, the three regions need to meet the following conditions:

 region A:
  (13) $$\begin{array}{c} R_{0}^{2}>1,\;R_{0}^{3}>1⟺p<\frac{1}{\left(10.7\left(1-\beta_{1}\right)\right)}, \end{array}$$
 region B:
  (14) $$\begin{array}{c} R_{0}^{2}>1,\;R_{0}^{3}<1⟺p>\frac{1}{\left(10.7\left(1-\beta_{1}\right)\right)},\\ \beta_{1}>\frac{\left(0.469\left(p^{2}-0.081p+0.008\right)\left(p-0.091\right)\right)}{\left(\left(p+0.0035\right)p\right)}, \end{array}$$
 region C:
  (15) R01<1, R02<1⟺β1<0.469p2−0.081p+0.008p−0.091p+0.0035p.

At the same time, the divide lines of the three regions can be calculated. Divide line of region A and region B:

(16) R03=1⟺β1=(0.469(p2−0.081p+0.008)(p−0.091))((p+0.0035)p).

Divide line of region B and region C:

(17) $$\begin{array}{c} R_{0}^{2}=1⟺p=\frac{1}{\left(10.7\left(1-\beta_{1}\right)\right)}. \end{array}$$

## 6. Discussion

Three steady states were obtained from the model described in this paper: in the first state, all populations coexist in the system; in the second state, there are no susceptible humans in the system, but the others coexist; and in the third state, only food animals remain in the system. Among these cases, only the first is a favorable outcome for humans. As shown in Figure 4, for a fixed *p*, as the value of *β*_1_ gets bigger, the possibility of coexistence will be smaller, even becoming impossible. Because the predation rate *p* cannot be easy controlled, in order to achieve the first state, we clearly need to reduce the value of *β*_1_.

On the other hand, the recent increase in society's attention to food safety suggests that food animals that consume antibiotic-containing feed might be consumed less by humans, which would result in a decrease in the predation rate *p*. At this point, to achieve coexistence, a smaller value of *β*_1_ is needed. Therefore, proper control of the amount of feed antibiotics used is necessary.

How can the value of *β*_1_ be reasonably controlled? When *R*_0_^2^ > 1 and *R*_0_^3^ > 1, the four species can coexist, and we obtain the relationship between *β*_1_ and *p* shown in Section 5.2. Therefore, we can adjust the value of *β*_1_ to satisfy the condition of coexistence.

As the value of *p* is not easy to measure and control, we instead need to focus on controlling the amount of antibiotics in the feed. As the value of *p* is expected to decrease with increased public awareness of food safety, controlling the value of *β*_1_ will become increasingly important. If we ignore the amount of antibiotics used in feed, and the predation rate continues to decrease, the antibiotic use rate could potentially surpass our “safety margin” without notice. In such a scenario, the human population will be predominantly susceptible and could become extinct in the most extreme case. In order to avoid this situation, proper control of the amount of antibiotics used in feed for food animals is extremely necessary.

## Figures and Tables

**Table 1:** Asymptotic states for all equilibrium points.

Equilibrium point	Feasible conditions	Dynamic behavior
*E*_1_	*R*_0_^1^ < 1; *R*_0_^2^ < 1	Two types of predator become extinct
*E*_2_	*R*_0_^2^ > 1; *R*_0_^3^ < 1	Susceptible person becomes extinct
*E*_3_	*R*_0_^2^ > 1; *R*_0_^3^ > 1	All species coexist

**Table 2:** Simulation results for fixed parameters: *b* = 0.8, *K* = 15, *p* = 0.45, *d*_1_ = 0.02, *β*_2_ = 0.005, *e* = 0.05, *d*_2_ = 0.07, *d* = 0.01, and *β*_3_ = 0.08.

Reproduction numbers' range	*β*_1_ varied in a range	Stable equilibrium point	Dynamic behavior
*R*_0_^1^ < 1; *R*_0_^2^ < 1	0.79 < *β*_1_ < 1	*E*_1_	Both predators become extinct
*R*_0_^2^ > 1; *R*_0_^3^ < 1	0.14 < *β*_1_ < 0.79	*E*_2_	Susceptible predators become extinct
*R*_0_^2^ > 1; *R*_0_^3^ > 1	0 < *β*_1_ < 0.14	*E*_3_	All the populations coexist

**Table 3:** Simulation results for fixed parameters: *b* = 0.8, *K* = 15, *β*_1_ = 0.1, *d*_1_ = 0.02, *β*_2_ = 0.005, *e* = 0.05, *d*_2_ = 0.07, *d* = 0.01, and *β*_3_ = 0.08.

Reproduction numbers' range	*p* varied in a range	Stable equilibrium point	Dynamic behavior
*R*_0_^1^ < 1; *R*_0_^2^ < 1	0 < *p* < 0.104	*E*_1_	Both predators become extinct
*R*_0_^2^ > 1; *R*_0_^3^ < 1	0.104 < *p* < 0.351	*E*_2_	Susceptible predators become extinct
*R*_0_^2^ > 1; *R*_0_^3^ > 1	0.351 < *p* < 1	*E*_3_	All the populations coexist

## Appendices

### A. Proof of Theorem 2

Proof Let

(A.1) R01=epK1−β1d+bβ1dd1+d1bβ1+β3bβ1K−β3bβ12K,R02=epK1−β1d2.

For the equilibrium point *E*_1_(*Kd*(1 − *β*_1_)/(*d* + *bβ*_1_), *Kβ*_1_ *b*(1 − *β*_1_)/(*d* + *bβ*_1_), 0,0), the Jacobi matrix is

(A.2) $$\begin{array}{c} J_{1}=\left[\begin{array}{cccc} \frac{bd\left(\beta_{1}-1\right)}{\left(d+b\beta_{1}\right)} & \frac{bd\left(\beta_{1}-1\right)}{\left(d+b\beta_{1}\right)} & \frac{pKd\left(\beta_{1}-1\right)}{\left(d+b\beta_{1}\right)} & \frac{pKd\left(\beta_{1}-1\right)}{\left(d+b\beta_{1}\right)}\\ b\beta_{1} & -d & \frac{pb\beta_{1}K\left(\beta_{1}-1\right)}{\left(d+b\beta_{1}\right)} & \frac{pb\beta_{1}K\left(\beta_{1}-1\right)}{\left(d+b\beta_{1}\right)}\\ 0 & 0 & J_{33} & 0\\ 0 & 0 & \frac{\beta_{1}b\beta_{3}K\left(1-\beta_{1}\right)}{\left(d+b\beta_{1}\right)} & -d_{2}+Kep-ep\beta_{1}K \end{array}\right], \end{array}$$

where  *J*_33_ = −(*dd*_1_ + *d*_1_ *bβ*_1_ − *epKd* + *epKdβ*_1_ − *epbβ*_1_ *K* + *epbβ*_1_^2^ *K* + *β*_3_ *bβ*_1_ *K* − *β*_3_ *bβ*_1_^2^ *K*)/(*d* + *bβ*_1_). The eigenvalues of *J*_1_ are

(A.3) λ1=−dd1+d1bβ1−epKd+epKdβ1−epbβ1KX22hhhhhhh+epbβ12K+β3bβ1K−β3bβ12KX22hhhh·d+bβ1−1λ2=−d2+Kep−epβ1Kλ3=−bd−d2+b2d2−2bd3+d4X22hhhhhhhhhhhhhhh+4bd3β1+8b2β12d2hhhhhhhhhhhhhhh−8b2β1d2+4b3β13d−4b3β12d1/2,  ·2d+bβ1−1λ4=−bd−d2−+4b3β13d−4b3β12d1/2b2d2−2bd3+d4+4bd3β1hhhhhhhhhhhhhh+8b2β12d2−8b2β1d2hhhhhhhhhhhhhh+4b3β13d−4b3β12d1/2  ·2d+bβ1−1.

According to *R*_0_^1^ < 1, *R*_0_^2^ < 1, we get *λ*_1_ < 0, *λ*_2_ < 0, and

(A.4) bd+d22−b2d2−2bd3+d4+4bd3β1+8b2β12d2hhhhhhhhhhhh−8b2β1d2+4b3β13d−4b3β12d  =4bd(1−β1)d+bβ12>0.

The real parts of *λ*_3_, *λ*_4_ are both negative. Using the Routh-Hurwitz criteria, the equilibrium point *E*_1_(*Kd*(1 − *β*_1_)/(*d* + *bβ*_1_), *Kbβ*_1_(1 − *β*_1_)/(*d* + *bβ*_1_), 0,0) is locally asymptotically stable.

### B. Proof of Theorem 3

Proof For the equilibrium point $E_{2}({\tilde{x}}_{1},{\tilde{x}}_{2},0,{\tilde{y}}_{2})$, where

(B.1) x~1=d2Kepd+Kepb−d2b−Kepbβ1epKepd+Kepb−d2b,x~2=Kβ1d2bKepd+Kepb−d2b,y~2=b(Kep−Kepβ1−d2)(ep2K).

For the convenient expression, let

(B.2) A=Kepd+Kepb−d2b,B=Kepd+Kepb−d2b−Kepbβ1,C=bKep−Kepβ1−d2,M=A(KepC+Bb)>0,M1=ACβ2+β3K2β1d2bep2−d2−d1Aep2K.

So the equilibrium point and reproduction number can be expressed by

(B.3) $$\begin{array}{c} {\tilde{x}}_{1}=\frac{d_{2}B}{\left(Aep\right)},\;\;{\tilde{x}}_{2}=\frac{K\beta_{1}d_{2}b}{A},\\ {\tilde{y}}_{2}=\frac{C}{\left(ep^{2}K\right)},\\ R_{0}^{3}=\frac{d_{2}Aep^{2}K}{\left(AC\beta_{2}+\beta_{3}K^{2}\beta_{1}d_{2}bep^{2}+d_{1}Aep^{2}K\right)}. \end{array}$$

Because of *R*_0_^3^ < 1, it is easy to get *M*_1_ > 0. The Jacobi matrix is

(B.4) $$\begin{array}{c} J_{2}=\left[\begin{array}{cccc} -\frac{bd_{2}B}{AepK} & -\frac{bd_{2}B}{AepK} & -\frac{d_{2}B}{Ae} & -\frac{d_{2}B}{Ae}\\ b\beta_{1} & -\frac{\left(C+Kepd\right)}{Kep} & -\frac{pK\beta_{1}d_{2}b}{A} & -\frac{pK\beta_{1}d_{2}b}{A}\\ 0 & 0 & -\frac{\left(AC\beta_{2}+\beta_{3}K^{2}\beta_{1}d_{2}bep^{2}\right)}{\left(ep^{2}KA\right)}+d_{2}-d_{1} & 0\\ \frac{C}{pK} & \frac{C}{pK} & \frac{\left(AC\beta_{2}+\beta_{3}K^{2}\beta_{1}d_{2}bep^{2}\right)}{\left(ep^{2}KA\right)} & 0 \end{array}\right]. \end{array}$$

The characteristic equation is

(B.5) $$\begin{array}{c} \lambda^{4}+A_{1}\lambda^{3}+A_{2}\lambda^{2}+A_{3}\lambda +A_{4}=0, \end{array}$$

where

(B.6) A1=M1+Kep2Bb+dAep2K>0,A2=M1BKepb+d+pd2MAK2e2p3A2>0,A3=d2M1M+A2BCep2KK3e3p4A2>0,A4=M1ABCd2K3e3p4A2>0,A1A2−A3 =(b+d)X22   ·(M1Kep2Bb+M12+p2d2MA+M1Kep2Bd)   X22+A2p2d2bd2C+b+dBbhhhh·K2e2p4A3−1>0,A1A2A3−A32−A12A4 =d2BAbd2C+b+dBbhhh·d2Bep4KCA3+M1d2Mp2A+M13hhhhh+M12BKep2d+M12BKebp2hhh·K5e5p8A5−1>0.

Using the Routh-Hurwitz criteria, the equilibrium point $E_{2}({\tilde{x}}_{1},{\tilde{x}}_{2},0,{\tilde{y}}_{2})$ is locally asymptotically stable.

### C. Proof of Theorem 4

Proof If a periodic orbit of model (1) in *Ω* exists, its projection onto some two-dimensional subspace of *R* ^4^ should also be periodic. Therefore, we have to investigate if any periodic solution exists or not an all two-dimensional subspace. There are six different two-dimensional subsystems of (1). For the subsystem,

(C.1) x˙1=bx1(1−x1+x2K)−bβ1x1−px1y1−px1y2,x˙2=bβ1x1−px2y1−px2y2−dx2.

We choose the Dulac function *B*_1_ = 1/*x*_1_ *x*_2_, to evaluate the following expression:

(C.2) $$\begin{array}{c} \frac{\partial \left(B_{1}{\dot{x}}_{1}\right)}{\partial x_{1}}+\frac{\partial \left(B_{1}{\dot{x}}_{2}\right)}{\partial x_{2}}=-\frac{b}{Kx_{2}}-\frac{b\beta_{1}}{x_{2}^{2}}<0. \end{array}$$

For the other five subsystems, we choose the Dulac functions

(C.3) B2=1x2y1,  B3=1y1y2,  B4=1x1y1,B5=1x1y2,  B6=1x2y2;

similarly

(C.4) $$\begin{array}{c} \frac{\partial \left(B_{2}{\dot{x}}_{2}\right)}{\partial x_{2}}+\frac{\partial \left(B_{2}{\dot{y}}_{1}\right)}{\partial y_{1}}=-\frac{b\beta_{1}x_{1}}{x_{2}^{2}y_{1}}<0,\\ \frac{\partial \left(B_{3}{\dot{y}}_{1}\right)}{\partial y_{1}}+\frac{\partial \left(B_{3}{\dot{y}}_{2}\right)}{\partial y_{2}}=-\frac{\beta_{3}x_{2}}{y_{2}^{2}}<0,\\ \frac{\partial \left(B_{4}{\dot{x}}_{1}\right)}{\partial x_{1}}+\frac{\partial \left(B_{4}{\dot{y}}_{1}\right)}{\partial y_{1}}=-\frac{b}{Ky_{1}}<0,\\ \frac{\partial \left(B_{5}{\dot{x}}_{1}\right)}{\partial x_{1}}+\frac{\partial \left(B_{5}{\dot{y}}_{2}\right)}{\partial y_{2}}=-\frac{b}{Ky_{2}}-\frac{\beta_{3}x_{2}y_{1}}{x_{1}y_{2}^{2}}<0,\\ \frac{\partial \left(B_{6}{\dot{x}}_{2}\right)}{\partial x_{2}}+\frac{\partial \left(B_{6}{\dot{y}}_{2}\right)}{\partial y_{2}}=-\frac{b\beta_{1}x_{1}}{x_{2}^{2}y_{2}}-\frac{\beta_{3}y_{1}}{x_{2}^{2}}<0. \end{array}$$

Now, using the Bendixson-Dulac negative criterion, no periodic solution in these two dimensions can exist. Therefore, the solution of (1) in *R* ^4^ also cannot oscillate persistently.
